# Designing Virtual Reality–Based Conversational Agents to Train Clinicians in Verbal De-escalation Skills: Exploratory Usability Study

**DOI:** 10.2196/38669

**Published:** 2022-07-06

**Authors:** Nathan Moore, Naseem Ahmadpour, Martin Brown, Philip Poronnik, Jennifer Davids

**Affiliations:** 1 Digital Health Solutions Western Sydney Local Health District North Parramatta Australia; 2 Design Lab Sydney School of Architecture, Design and Planning The University of Sydney Sydney Australia; 3 Faculty of Medicine and Health Media Lab, Education Innovation, School of Medical Sciences Faculty of Medicine and Health The University of Sydney Sydney Australia; 4 Research and Education Network Western Sydney Local Health District Westmead Australia

**Keywords:** virtual reality, code black, verbal de-escalation, violence and aggression, education, clinical training, conversational agent

## Abstract

**Background:**

Violence and aggression are significant workplace challenges faced by clinicians worldwide. Traditional methods of training consist of “on-the-job learning” and role-play simulations. Although both approaches can result in improved skill levels, they are not without limitation. Interactive simulations using virtual reality (VR) can complement traditional training processes as a cost-effective, engaging, easily accessible, and flexible training tool.

**Objective:**

In this exploratory study, we aimed to determine the feasibility of and barriers to verbal engagement with a virtual agent in the context of the Code Black VR application. Code Black VR is a new interactive VR-based verbal de-escalation trainer that we developed based on the Clinical Training Through VR Design Framework.

**Methods:**

In total, 28 participants with varying clinical expertise from 4 local hospitals enrolled in the Western Sydney Local Health District Clinical Initiative Nurse program and Transition to Emergency Nursing Programs and participated in 1 of 5 workshops. They completed multiple playthroughs of the Code Black VR verbal de-escalation trainer application and verbally interacted with a virtual agent. We documented observations and poststudy reflection notes. After the playthroughs, the users completed the System Usability Scale and provided written comments on their experience. A thematic analysis was conducted on the results. Data were also obtained through the application itself, which also recorded the total interactions and successfully completed interactions.

**Results:**

The Code Black VR verbal de-escalation training application was well received. The findings reinforced the factors in the existing design framework and identified 3 new factors—motion sickness, perceived value, and privacy—to be considered for future application development.

**Conclusions:**

Verbal interaction with a virtual agent is feasible for training staff in verbal de-escalation skills. It is an effective medium to supplement clinician training in verbal de-escalation skills. We provide broader design considerations to guide further developments in this area.

## Introduction

### Background

Violence and aggression in health care settings is an international problem, with one-fifth of health care professionals experiencing violence perpetrated by patients or family members every year [1]. The implementation of effective training in high-quality verbal de-escalation skills has been shown to be vital for the well-being of staff and patients. When used early, these skills can prevent escalation of the situation to violence [2].

“A Code Black is any incident where hospital staff are threatened with or experience verbal, physical or psychological abuse or attack during the course of their employment and a staff response is required” [3]. The code black response involves a variety of interventions that may be required preincident occurrence, during the incident, or postincident occurrence [3]. There is evidence that the delivery of high-quality violence and aggression minimization training has a positive impact on staff member’s perceived confidence in managing these situations [4]. In addition, this style of training corelates with a reduction in the number of incidents of violence and aggression occurring in health care [5].

Current practice for Violence Prevention and Management training within our Local Health District (LHD) is conducted using a traditional face-to-face didactic simulation and skills station format. This training allows clinicians to collaborate and practice these skills in a group setting with expert support from course instructors. Simulation has been clearly established as an effective way to train clinicians in the application of skills and to reduce anxiety before the application of these skills [6]. However, a significant challenge posed by this type of training is that it requires significant resources and clinicians to be present in a fixed location and time [7]. Given the resource limitations, the complexity of staffing requirements, and the high number of staff members requiring training, the current demand for this training far exceeds the capacity for the program’s delivery. In addition, there is a need for refresher training for staff, and this is not factored into the current program’s face-to-face delivery schedule. e-Learning training modules and videos are available; however, there are questions about the efficacy of e-learning to impact health care professional’s behaviors, skills, or knowledge [8].

Owing to these challenges and the increasing occurrence of code black, we developed a pilot supplemental virtual reality (VR)–based application to support clinician education in code black management. The initial phase of this project scoped the breadth of the problem and involved staff interviews and incident review. Our initial findings highlighted that staff required training on how to recognize behaviors of concern in an individual and practice in verbal de-escalation skills to minimize or prevent aggression or violence toward a staff member [2]. Therefore, we reasoned that there is a need for readily accessible and frequently repeatable experiential training in managing code black events [3].

To address the barriers of access, cost, and availability of training, VR is being adopted as a supplemental modality to support clinician education in several scenarios [9]. For example, VR has been explored to train students in conflict management as part of the research being conducted by the University of Newcastle [10]. While still in trial, the approach is being well received by students and is showing potential for providing education in this challenging area. Previously, we used VR as a tool within the health district to train clinicians in the management of advanced life support (ALS) [11]. Building upon our experience in this area and our findings in previous VR user needs analysis [12], we developed a VR-based application to support code black training.

### VR Technology for Verbal De-escalation Training

The novel capabilities of VR provide significant potential for educators to use this approach to supplement and, in some cases, replace traditional learning modalities [13,14]. The interaction with 3D representations of people, items, and environments in real time can allow users to practice the application of skills. Deploying a VR app on portable and standalone VR head-mounted displays (HMDs) enables users to engage with training in a time and setting of their choosing, which may not have been possible otherwise [9,15]. In addition, the level of *blocking out* of the physical world made possible by the VR HMDs can further increase the immersion of the user and increase presence; that is, the feeling of being “present” in the scenario [16]. This results in the suspension of disbelief and the generation of authentic reactions in users due to increased engagement with the experience. The ability to record and assess user interactions within a VR app allows for completely objective, structured assessment and feedback on the platform, which can be difficult with human assessors [17]. Voice recognition within the application can add an element of increased engagement with the technology but also brings with it challenges and limitations with regard to misinterpretation or misunderstanding errors, which must be overcome [18].

### Conversational Agents for Training

Increasing processor power and technological advancements have resulted in the emergence of virtual conversational agents as a potential training modality. BodySwaps is a company developing VR-based “soft skills” training where users can observe interactions between virtual agents and make structured observations and interact with the virtual agent to provide comment and feedback [19]. The responses are recorded and provided as feedback to the user for development. The content provided to the users focuses on human resources and management-style scenarios. Other studies using virtual conversational agents have been conducted by the University of Newcastle in an application built to train student nurses called “Angry Stan.” The application uses biofeedback to train clinicians to remain calm in confronting situations. The user responses are chosen from a multiple-choice list of scripted responses, and the agent responds to both the choices made by the user and their chosen responses [20].

### Objectives

It is clearly identified that a structured and coordinated response by well-trained staff using a shared organizational approach is vital to minimize the risk to both staff and patients [21]. Given the identified barriers to the accessibility of existing training approaches, we aimed to develop a VR-based supplemental code black training application. We have previously developed VR-based applications to support clinical education in areas such as ALS management, leadership, clinical handover, and dignity in the workplace [11]. However, verbal interaction with a virtual agent that can respond to user input is a far more complex challenge, and there is little research that can guide the design of such interactions.

The aim of this study was 2-fold: (1) to determine the validity of using the existing “Clinical Training Through VR Design Framework” to assess the feasibility of VR-based education modules [12] and (2) to identify specific design requirements for a VR-based agent to train clinicians in verbal de-escalation skills in the context of code black management.

The Clinical Training Through VR Design Framework involves 8 factors that define the clinician training needs that must be supported within the VR environment and through the interactions [12]. These factors are realistic tasks, visibility, completion, accessibility, agency, diverse input modalities, mental models, and advanced roles [12]. In this original iteration of the framework, a subgrouping of some commonly associated factors was provided.

## Methods

### Overview

This was a mixed methods study. Opportunistic recruitment occurred during the Western Sydney LHD (WSLHD) Transition to Emergency Nursing and Clinical Initiative Nurse programs. Five workshops were conducted during these programs, each lasting approximately 20 minutes at the Westmead Hospital Simulated Learning Environment for Clinical Training simulation center. Data were collected through qualitative observation notes by researchers and a postuse survey was completed by participants, which were put into the context of analytic data obtained from the Code Black VR system.

At the start of the study, participants were given an orientation that consisted of a brief overview of the study and instructions regarding the use of the Oculus Quest 2 headset. Following the orientation, the participants undertook multiple unguided playthroughs in VR with instructors (NM, MB, and JD) observing the group and answering any questions. Each individual playthrough lasted approximately 10 minutes. Upon completion, the participants completed a questionnaire based on the System Usability Scale (SUS) [22] and had the opportunity to add open-ended comments at the end. The SUS was used for this study because it is a widely adopted tool for the evaluation of user interfaces with a focus on usability [23]. The SUS consists of 10 distinct questions designed to evaluate the user interface. Each question was rated on a 5-point scale ranging from strongly agree to strongly disagree. The final calculation provides a score of 0 to 100 with a SUS score of more than 68 being deemed “above average” [22]:

1. I think that I would like to use this system frequently.
2. I found the system unnecessarily complex.
3. I thought the system was easy to use.
4. I think that I would need the support of a technical person to be able to use this system.
5. I found the various functions of this system were well integrated.
6. I thought there was too much inconsistency in this system.
7. I would imagine that most people would learn to use this system very quickly.
8. I found the system very cumbersome to use.
9. I felt very confident using the system.
10. I needed to learn a lot of things before I could get going with the system.

The researchers present during the session also documented their reflections on the overall session after the workshops. The data obtained from the SUS were consolidated, and a final score was calculated to determine the overall usability of the system. A thematic analysis was conducted on participants’ open-ended comments to identify common themes and better understand the user experience of the tool. Researcher observations were summarized separately, offering insights into the identified themes. In addition, the back-end application records the success and failure of speech-to-text (STT) interpretations. These analytics were collated to provide objective data on in-app use.

### Code Black VR

#### Design Rationale

On the basis of prior experience with developing simulation-based educational experiences in both traditional and virtual settings, we identified that any solution to support this training needed to be flexible and must address the needs of both clinicians and educators alike. In our view, to be truly flexible, the VR solution had to run on portable and untethered HMDs. This allows “free range” deployment into any environment as there is no need for any additional external computers.

To address the requirement of verbal de-escalation skill training, a realistic dialogue between the user and the virtual agent is vital. In collaboration with Frameless Interactive, we used microphone access within the applications for the Oculus Quest 2 HMD. Microphone access allows voice-to-speech transcription to occur within the VR application. Keyword analysis and categorization of the resulting text file enables the app linguistic program to produce text-based responses that are then converted by text-to-speech software using the Google text-to-speech application programming interface into credible verbal interaction with a virtual agent within the HMD. Using the 8 preidentified affordances is vital for VR design for clinician education, and this guided the initial prototype build of the Code Black VR verbal de-escalation trainer that was tested in this study.

The application was created to work on the Oculus Quest 2 HMD [24]. The portable nature, processing power, and microphone capability of the Quest 2 makes it an ideal choice for this application. The Code Black VR verbal de-escalation training application was built on the Unreal game engine [25]. Unreal was used because it has high-level textures and editing capabilities and provides a finished product of a high visual standard. In addition, we were able to leverage developments and learnings from the ALS-SimVR [11] app to improve the cost-effectiveness of the development process.

#### Verbal De-escalation Trainer Walkthrough

**Figure 1:**
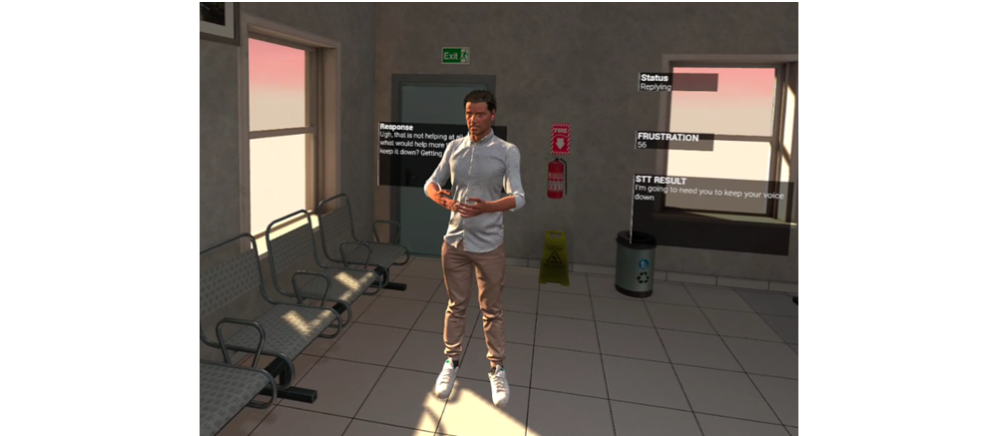
Virtual conversational agent with status, frustration, response, and speech-to-text result.

The interactive simulation positions the user in an emergency department (ED) waiting room in front of a visibly distressed digital conversational agent named Louis. The man identifies himself as the son of a patient who is awaiting review by a physician. The man is distressed by how long they have been waiting. The user who was an ED nurse, was instructed during the orientation to press the “Y” or “B” buttons on the Quest 2 controller using a push-to-talk approach to verbally respond to the patient. Following the user response, the agent responds in either a positive or negative fashion, depending on the input. Responses categorized as compassionate resulted in a decrease in the agent’s level of aggression; responses categorized as confrontational resulted in an increase in the agent’s level of aggression. The responses also drive an increase or decrease in the background “frustration level,” which controls the agent’s animations and subsequent responses. In the prototype build, the user can also see information about the progress of the scenario, such as the current “frustration level,” recorded STT results, and agent responses (Figure 1).

**Figure 2:**
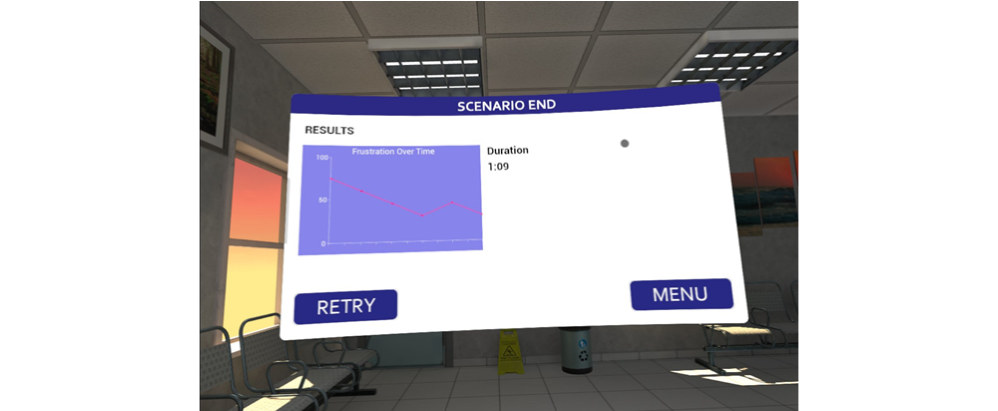
User feedback after the scenario.

Following a series of verbal interactions or a set period, the user will have either increased or decreased the agent’s frustration level, and the scenario will conclude depending on what the user said and how they responded. Following the end of the scenario, the user is presented with their performance data (Figure 2). These data are also recorded on the SimDash Learning Management System created by Frameless Interactive to support all of our VR-based applications. This learning management system allows for more in-depth feedback. The user can later log in to review their performance over time.

### Overview of Code Black VR: Verbal De-escalation Training Application Architecture

**Figure 3:**
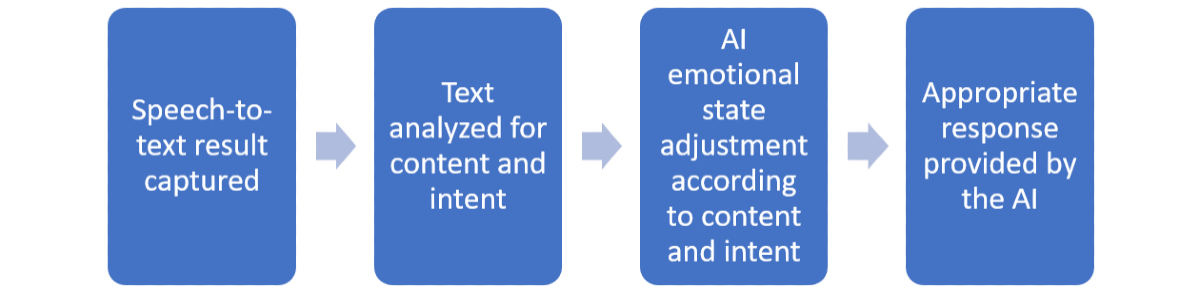
Artificial intelligence (AI) conversation flow.

The application places the user in a medical setting with proprietary emotional, conversational artificial intelligence (AI) called DriftAI, which drives an agent’s animation and voice. At the start of the scenario, the “frustration level” of the AI, as represented by the digital agent, approaches an angry state, and the user must attempt to de-escalate the agent. The emotional state of the agent shifts depending on what the user says. The AI captures the user’s speech input and analyzes the content of the conversation to determine the user’s intent. It then adjusts the mood of the agent and selects an appropriate response based on the agent’s emotional state for that intent (Figure 3).

The AI response is generated in several ways, from an audio source with associated facial motion capture, AI-generated voice with lip sync, or only text. This choice depends on whether the generated response is already “known” by the system. The dialogue tree is structured in a “sandbox” format. This provides the advantage of allowing any intent to be matched at any time, reducing the need for a large number of predetermined intents to be in place. The scenario runs on a strict input and response system. The story will only advance each time the user inputs something and the agent responds to it. This is repeated until an end point is reached, such as the agent is too angry or the user has successfully de-escalated the agent.

### Ethics Approval

This study was approved by the WSLHD Human Research Ethics Committee (2019/ETH00598). All the participants provided written informed consent.

## Results

### Overview

In total, 28 (19 female and 9 male) participants involved in the WSLHD Clinical Initiative Nurse program from 4 local hospitals participated in this study. Some participants were clinicians and educators (n=6) involved in the delivery of the program while others were junior to intermediate clinicians who were course participants (n=22). All participants had varied levels of experience working in LHD EDs, and the prerequisite of participation in the Clinical Initiative Nurse or Transition to Emergency Nursing Programs is some level of ED experience and exposure to aggression.

### SUS Scoring

All collected SUS data were analyzed according to the SUS scoring procedure of adding all odd-numbered questions and subtracting 5 to obtain X, adding all even and subtracting the total from 25 to obtain Y, then adding X by Y and multiplying by 2.5 [22]. Where there was a missing response, the average of the other responses was used in the calculations. Clinicians and education groups were reported separately to aid in identifying any differences in perspective regarding perceived usability. The scores are listed in Table 1.

**Table 1:** System Usability Scale (SUS) scoring of the participants (N=28).

Participant	Q^a^1	Q2	Q3	Q4	Q5	Q6	Q7	Q8	Q9	Q10	SUS (×2.5)^b^
Clinician 1	2	4	2	4	1	0	2	4	2	4	62.5
Clinician 2	2	1	2	2	1	2	2	2	1	2	42.5
Clinician 3	3	4	3	4	3	4	3	1	3	4	42.5
Clinician 4	2	4	4	1	4	2	4	4	4	4	82.5
Clinician 5	3	4	4	1	3	1	3	1	3	3	65
Clinician 6	2	3	2	1	1	0	1	2	1	3	40
Clinician 7	2	2	3	1	2	3	3	1	3	3	57.5
Clinician 8	3	4	4	1	2	2	4	1	2	3	65
Clinician 9	3	4	0	4	3	3	4	4	3	4	80
Clinician 10	3	3	2	4	4	2	3	2	1	2	65
Clinician 11	2	2	1	0	3	DNC^c^	3	1	1	1	67^d^
Clinician 12	3	1	3	1	3	3	3	2	1	1	52.5
Clinician 13	1	4	DNC	4	2	3	3	3	2	4	72^d^
Clinician 14	4	4	4	0	3	3	4	0	4	4	75
Clinician 15	4	1	3	0	2	2	4	3	2	2	57.5
Clinician 16	3	3	4	1	2	3	4	3	2	3	65
Clinician 17	3	2	3	3	2	1	3	4	4	3	70
Clinician 18	3	4	3	3	2	2	3	3	3	3	72.5
Clinician 19	3	3	3	2	4	1	4	3	4	3	75
Clinician 20	4	2	3	0	4	2	4	1	4	2	65
Clinician 21	3	3	2	0	3	2	2	1	2	1	47.5
Clinician 22	2	1	2	2	2	2	3	2	1	1	45
Education 1	2	3	2	0	1	2	2	2	1	1	40
Education 2	1	3	2	2	1	1	2	3	1	3	47.5
Education 3	1	3	3	2	2	3	3	3	2	4	65
Education 4	1	3	1	2	2	0	1	3	DNC	3	44^d^
Education 5	3	3	3	0	2	3	3	4	3	2	65
Education 6	3	2	2	1	2	1	3	2	1	0	42.5

^a^Q: question.

^b^Average usability: 59.5.

^c^DNC: did not complete.

^d^Average of results used to calculate total.

### Thematic Analysis

Three researchers (NM, MB, and JD; all experienced in educational VR development) performed a thematic analysis [26]. Owing to the clinical skill overlap between the clinical and educational participants, we decided to conduct the thematic analysis on the combined data. After coding, the comments were grouped based on the factors defined in the design framework reported in our previous study [12]. The factors identified in the previous study helped inform the development of this application and as such were relevant to the analysis of the newly gathered data [12]. The data were then reviewed using an inductive (bottom-up) approach following the process of reflexive thematic analysis (Braun and Clarke [26]) to identify any potential codes and themes that may fall outside the initial design framework.

This process helped determine whether design considerations made more broadly for VR clinical training applications correlate with participant experiences in Code Black VR given that this app uses verbal interactions with a conversational agent. Participants provided insights and comments that specifically corresponded to the 8 factors defined in our framework. However, we also identified 3 other factors that were salient in relation to Code Black VR specifically, namely motion sickness, perceived value, and privacy. For clarity, we have removed the subcategory of affordances. All factors and exemplar descriptions are summarized in Textbox 1.

**The revised Clinical Training Through Virtual Reality Design Framework with 11 factors guiding the experience design and exemplar statements.**

**Factors and exemplar statements**

- Advanced roles
  The ability to manage tasks at an acceptable standard
- Accessibility
  Clarity as to how commands are given and accessed
- Agency
  The environment providing opportunity to control workflows autonomously and make choices that align with prior experiences, such as multitasking
- Completion
  Clear commencement and completion prompt to task
- Diverse input modalities
  The environment replicates natural input modalities such as issuing commands verbally
- Mental models
  The environment design and prompts align with how the clinical environment operates (eg, 2 minutes of cardiopulmonary resuscitation completion)
- Motion sickness
  That all efforts are made to reduce the experience of motion sickness for the user so they can engage with the experience
- Perceived value
  The application provides an experience perceived as valuable by the user
- Privacy
  The application and deployment experience should maintain the user’s privacy
- Realistic tasks
  Common clinical tasks should be available for completion in a realistic manner
- Visibility
  Clear visible assets aligned with environmental orientation

### Factors

#### Advanced Roles

Many participants, particularly from the educator group, identified limitations in the way the application handled some of the “higher order” or “advanced skills” they would bring to a de-escalation situation. These included considerations of physical proximity, long-form verbal responses (which were often not interpreted accurately by the application), and the ability to bring the agent’s father into the discussion.

#### Accessibility

Participants highlighted the need for a more comprehensive orientation as to “what to do” and that it was “a bit overwhelming for a first timer.” One participant provided feedback, “I would need more education on how to work it.” Participants expressed preferences for the verbal interaction approach, but the “push-to-talk” method of interacting seemed to be counterintuitive or was forgotten by some participants during scenario playthroughs.

#### Agency

Participants wanted the ability to control and perform the required actions based on their personal experience. In the context of verbal de-escalation in health care, this poses a technological challenge. The often-lengthy verbal responses provided by clinicians were challenging for the AI to understand. This resulted in situations where participants stated, “the AI didn’t respond appropriately to a lot of my responses.” This often resulted in both a failure to detect what was captured accurately and interpret it correctly.

#### Completion

Lessons learned from our previous work in VR, which at times was unclear when the given experience was completed, informed the researchers to ensure a clear distinction for the participants as to when the verbal de-escalation scenario had finished. At the completion of the scenario, the participants were presented with feedback on their performance. They were then given the opportunity to restart the scenario and exit the application.

#### Diverse Input Modalities

Given the nature of verbal de-escalation skills requiring verbal interactions, participants found verbal interactions with the agent to be an important aspect of the application. Challenges were noted at times with inaccurate interpretations of what the participants had said by the application. Other input modalities, such as reducing proximity to the patient as a de-escalation tool, were also highlighted by the participants.

#### Mental Models

Several participants, particularly from the educator group, commented that the approach they were required to take for success within the application did not align with the approach they would take in the clinical environment. As noted in the *Advanced Roles* section, clinicians will use a variety of strategies to de-escalate a situation. The requests for response to “proximity” and being able to “utilise the dad to de-escalate the situation,” all spoke to the need for greater representation of participants’ mental models, which can be addressed in future iterations of the application.

#### Motion Sickness

Of the 28 participants, 3 (10%) either had their experience cut short or impacted due to motion sickness. One responder stated, “made me feel dizzy and I could not continue,” and another stated, “I felt uneasy standing.”

#### Perceived Value

As adult learners, for clinicians to engage with any educational approach, they must perceive the experience to have value and provide motivation to learn [27]. The perceived value of the verbal de-escalator experienced was mixed within the group, from positive sentiments, “I think it is a very clever concept and could definitely help to improve aggression in a waiting room and to learn how to de-escalate the situation before it gets worse” and “I think and can see a system like this could help train staff with de-escalation,” to those who questioned its value over simulation or its value for money, “it seems like a very expensive educational tool.”

#### Privacy

Some participants expressed concerns around “privacy.” For some participants, being aware of the presence of colleagues around them participating in the same experience was confronting and challenging at times. One participant reported, “I freaked out not knowing who was near me and where,” and another stated, “It felt very disconcerting having other people in the room I could hear but not see.”

#### Realistic Tasks

The participants in our study identified a clear need to be able to complete verbal de-escalation in the virtual environment in a manner that replicated the clinical environment. These include moving further from or closer to the patient and using broad and complex language constructs. In addition, the use of emotional and facial cues from the agent were requested as they are key skills used in de-escalation.

#### Visibility

Participants highlighted, on multiple occasions, the need for increased visibility and realism of the agent they were de-escalating and the surrounding environments. For example, the facial expressions of the agent did not adequately reflect participants’ expectations of a verbally aggressive person. One clinician stated, “his expression and body language didn’t change.” This is largely a technological and budgetary issue.

### Observations

Observations made by the research team highlighted several barriers that inhibited the participants’ ability to reach the desired objectives within the application.

Despite initial concerns about the learning curve required to access the application, based on previous VR deployments, we noted that the participants seemed to orientate to the interaction within a few minutes and engage quite quickly and naturally with the virtual agent. We suggest that this was due to the simplicity of the design; that is, there were only 2 buttons on the controller required to engage with the application (front trigger for selection and the top Y or B button for push to talk) and then interact verbally, which is an inherent skill of all participants.

We noted comments from the participants requesting training in the skills required for de-escalation before using the application. We plan to implement a training mode within the application itself that would be completed before experiencing the simulation scenario. We initially assumed that participants would feel adequately prepared to respond to the situation in this setting. However, our observations have further reinforced the need to provide the opportunity for both tutorials on how to use the application and for training for the specific skills to be used before the virtual simulation within the application for use, if required. This lack of appropriate orientation affected the accessibility of the application.

As this is a beta pilot version, the application presents the participant with minimal preparation for the scenario. However, this setting represents a common and realistic situation faced by clinicians working in the ED. Nevertheless, it was clear that this was an issue for some participants who were seeking further information, such as the agent’s father’s condition or more background information normally available in the medical records.

### Analytics Data

A total of 79 scenarios were captured and uploaded to the database during the workshops. In some instances, some scenarios may not have been captured because of participant error or early disconnection. In the 79 scenarios, a total of 416 “interactions” with the AI occurred. Of these 416 interactions, 96 had an empty STT input, which indicates nonrecognition of what the participant had said. This may have occurred because of a misalignment between when the participant pressed the button to start recording and when they started verbally responding to the agent.

## Discussion

### Principal Findings

The results of this study provide novel insights into the potential benefits of using verbal interactions with a VR-based conversational agent to supplement traditional verbal de-escalation training. It has been shown that immersive high-fidelity simulation can be beneficial over participant-to-participant role play due to a higher level of authenticity presented to enable the learning of verbal skills [28]. However, to date, there is little evidence of how such simulations can be translated into virtual settings with an elevated level of accuracy. This is principally due to the technology still being in relative infancy as well as the core functionality of speech recognition having identified challenges for more specialized use [29].

We conducted usability testing with feedback collated from the SUS form and free-text comments, as well as detailed observations taken throughout the study. The findings indicated the feasibility of the 8 factors identified to be key to the experience of users in VR training applications [12]. This study can provide a useful design direction for Code Black VR and future VR applications for clinical training. The feedback also highlighted several factors that impacted the experience, specifically within the Code Black VR, which were not part of the existing framework, motion sickness, perceived value, and privacy. We believe that these findings were identified because new factors included in this study were not observed in our previous work. The larger number of participants increased the likelihood of having participants who experienced certain elements of motion sickness, which is a well-documented side effect for some users [30]. We believe the “perceived value” factor arose in this study as it speaks to the highly specialized context of this application. Verbal de-escalation skills are perceived as a vital skill by and for clinicians; however, there is no real standardized way in which the content is delivered. Traditionally, these are skills established over years of service with significant complexity and nuance in their execution [3]. The factor of privacy was an unexpected but valuable outcome from this study. In previous studies, authors had conducted VR-based trials with small groups or individual interventions. The nature of this study and the logistics of completing it during the available time slots meant that larger groups were undertaking the experiences in closer proximity. This resulted in an innate awareness of the proximity of others and highlighted the need for this to be considered in future deployment strategies.

The average usability score of 59.5 placed Code Black VR’s usability into the “poor” ranking on the usability scale. An SUS score below 68 indicates issues with design that require research and resolution [23]. This was not surprising, as the aim of this study was to test the feasibility of a newly developed prototype with significant scope for growth. The SUS tool helped the team identify areas of improvement to focus on in the next phase. One example was the need for improved orientation to the application; for example, through an interactive tutorial. We had acknowledged this need previously but underestimated its importance, given the perceived “natural verbal interactions” that the application was incorporating. This provides useful insight for future development and research in this area.

One surprising observation we made was the difference in perceived usability between the educator group (average score 50.6) and the clinician group (average score 61.6; Table 1). We suggest that this disparity arises from the different frames of reference of the more skilled and experienced clinicians and the perceived requirements of experience to train high-level verbal de-escalation skills. This was compared with the understanding of the requirements of the more junior group of clinicians in the study group. This finding aligns with our targeting of the application toward novice or intermediate entrants to the profession to develop basic levels of skills. The ultimate outcome is for high-level dialogue with a virtual agent; however, this requires significant further research and development of the AI system to reach that level of maturity, which is not achievable in the short term for these projects. Understanding the differences between the experience of the 2 groups provides a useful distinction for future studies on VR apps for clinical training and one that has not been addressed in the literature.

The analytics data allowed us to determine the number of completed scenarios and interactions and confirmed that the technology was functional. The empty STT inputs further reinforced the need to revise not only the way the user is orientated to the experience but also the way the interactions are captured within the device. When clinicians were engaged in dialogue, we witnessed several occasions where the clinicians “forgot” to use the push-to-talk function before responding to the agent. We are currently exploring the use of continual recording of user responses to minimize these dropouts; however, this brings different challenges, such as unintended responses, background noise, and misalignment with overlap occurring between the user and agent response cycles.

### Challenges and Opportunities in Verbal De-escalation Training in VR

The thematic analysis of the participant responses highlights potential opportunities and limitations for using a combination of VR and conversational agents to address the challenges of developing verbal de-escalation skills. We predict a rapid growth in the application of VR to supplement clinical education in the future, as evidenced by the number of innovations occurring in our LHD. Despite our previous work identifying “user needs” in the design of VR-based applications [11,12], we identified several challenges in applying our knowledge in the context of the Code Black VR application, because this application features a conversational agent. The complexities of human language with variations in sentence structure, accents, and training are challenging to accommodate in an AI setting [31]. Therefore, while we are attempting to enhance user agency and allowing for advanced roles to align with the clinician’s mental models, the technical limitations faced by the current build undermine both usability and user experience. We address some of the ways in which these factors could be addressed in the *Code Black VR—Verbal De-escalation Skills Trainer: Next Steps* section.

### Code Black VR—Verbal De-escalation Skills Trainer: Next Steps

This study identified several areas where Code Black VR can be improved to enhance the usability and user experience. The improvements and next steps are addressed through language recognition and generation, supplemental education, intra-application efficiencies, and broader application of code content.

### Language Recognition and Generation

We note that a next step will involve identifying how to interpret, more accurately, the long-form answers that clinicians were providing within their dialogue. This could be achieved by improving the conversation structure by using a staged diatribe approach. This approach will connect user intents together, tagging intents within contextual sections and bringing a more structured approach to the dialogue. This will allow for greater context and improved accuracy in the response system. This approach also coincides with the implementation of a keyword search through the multiple intents to further increase interpretation accuracy.

We also suggest implementing a process in which the user response recording is always being recorded and a few seconds of recording spoken before the interaction button press is considered in the submission to the AI. This implementation will assist the experience in 2 important ways. First, it provides a solution for late button presses when the user responds before pressing the interaction button. Second, the DriftAI system will interpret what the user is saying and compare this to previous responses to increase the accuracy of the STT interpretation and subsequent response.

Another next step will be to improve the way DriftAI handles repetition. Currently, the same response was observed to be triggered multiple times in a row. Even with broader dialogue options, this occurrence is still a possibility. The developers are working on a way to reduce the repetition by tagging some responses as “unrepeatable” to ensure it is only matched once.

Following the completion of the study reported in this paper, we have also begun trialing a more sophisticated “sandbox” mode to provide an even larger training library to the DriftAI system. This allows for a more realistic and open dialogue with the agent; however, more development is required to control the AI, so it is aware of the context and stays “on track” to meet the desired learning outcomes.

### Supplemental Education

The simulator, in its current state, requires clinicians to understand the basic verbal de-escalation skills to be successful. As such, we suggest implementing a module for novices to train them in these skills before using the simulator elements.

### Intra-Application Efficiencies

In conjunction with the verbal de-escalation trainer, we are also deploying several other VR-based applications to support code black training for clinicians. These applications use other VR-based methodologies, such as 360° video of immersive clinical scenarios, auto stitching of the different views to allow the randomization of the 360° videos, and interactive hot spots to use newly adopted observation charts. The objectives of these applications are to support other vital code black skills, such as situational awareness, team planning, the detection of early signs of escalation, role allocation, and observations using the Behaviours of Concern observation chart. The goal is to embed all these modules within a single interconnected training package to better support the needs of the target clinicians. When this occurs, further research of the educational outcomes and effectiveness of the complete package will be conducted.

### Broader Application of Core Content

We identified broader use cases for the underlying DriftAI technology that enables verbal interactions to occur. We will strive to use the findings of this study to adapt this verbal interaction approach to other clinical training situations using VR, flat-screen television, and mobile deployments.

### Limitations

The breadth of the participant group in our study was indicative of emergency staff experience and skill sets in our LHD. However, the Code Black VR app is planned to be used in future iterations outside the ED setting. As such, further testing with users not from an ED background will be essential for further development and deployment.

In addition, the build of the application tested was still in the preliminary stages of development. As such, there were limitations on the available responses of the agent and the ability of the STT system to interpret some of the longer responses that clinicians provided to the agent. It was an intentional decision to conduct the study on such an early build of the application to explore the feasibility of the approach, but future studies on the developed application will be required to better understand the user experience and potential educational outcomes.

Another limitation of this study was in the method of limiting data collection to written forms and observations. A greater depth of understanding would likely have been established with the inclusion of user interviews during the data collection phase. The reason this was not included is related to the availability of participants who were enrolled in the study on the condition that the study be accommodated into part of their training program, which did not leave adequate time for the interview. We plan to implement user interviews in later studies on further developed iterations of the application; it was unfortunately not feasible for this study.

We acknowledge that the novel nature of VR can result in the “novelty effect” [32], which could account for some of the findings. In addition, further research and comparative studies should be conducted to explore the sustained adoption and knowledge retention of such novel approaches.

### Conclusions

The implementation of effective verbal de-escalation training is essential to ensure the ongoing safety of both patients and health care providers. Simulation and face-to-face training are established approaches to deliver this type of training and increase staff confidence in these confronting situations. The challenge with the traditional approach of face-to-face simulation training is that it is asynchronous and resource intensive. The use of VR to supplement the existing training approaches is emerging as a feasible option. Our novel approach to using verbal interactions within VR was well received by clinicians. Our proposed framework with 11 factors could provide a much-needed direction for additional training assets to support clinicians faced with these challenging situations. The findings from this research contribute to the feasibility of our framework to support future research and the development of VR for clinical training. We have also contributed a set of requirements to guide the design of verbal interactions in the VR de-escalation training environment.

## Abbreviations

AI: artificial Intelligence
ALS: advanced life support
ED: emergency department
HMD: head-mounted display
LHD: Local Health District
STT: speech to text
SUS: System Usability Scale
VR: virtual reality
WSLHD: Western Sydney Local Health District
